# Improving Biodegradable Films from Corn Bran Arabinoxylan for Hydrophobic Material and Green Food Packaging

**DOI:** 10.3390/foods13121914

**Published:** 2024-06-18

**Authors:** Abdulrahman Alahmed, Senay Simsek

**Affiliations:** 1 Department of Food Science and Nutrition, College of Food and Agricultural Sciences, King Saud University, Riyadh 11451, Saudi Arabia; abalahmed@ksu.edu.sa; 2 Cereal Science Graduate Program, Peltier Complex, Department of Plant Sciences, North Dakota State University, Fargo, ND 58102, USA; 3 Whistler Center for Carbohydrate Research, Department of Food Science, Purdue University, West Lafayette, IN 47907, USA

**Keywords:** arabinoxylan, water barrier, film insolubility, hydrophobicity, biodegradability

## Abstract

Non-biodegradable plastic materials pose environmental hazards and contribute to pollution. Arabinoxylan (AX) films have been created for applications in food packaging to replace these materials. The water interaction characteristics of biodegradable AX films were assessed following the extraction of AX from dry-milled corn bran (DCB), wet-milled corn bran (WCB), and dried distiller’s grains with solubles (DDGS). Films were prepared with laccase and sorbitol before surface modification with lipase–vinyl acetate. Water solubility of the modified DCB films was significantly reduced (*p* < 0.05); however, the water solubility of modified WCB films decreased insignificantly (*p* > 0.05) compared to unmodified films. Water vapor permeability of the modified AX films from WCB and DDGS was significantly reduced (*p* < 0.05), unlike their unmodified counterparts. The biodegradation rates of the modified WCB AX and DDGS films increased after 63 and 99 days, respectively, compared to the unmodified films. The hydrophilic nature of AX polymers from WCB and DDGS enhances the biodegradability of the films. This study found that the modified WCB AX film was more hydrophobic, and the modified DDGS AX film was more biodegradable than the modified DCB AX film. Overall, surface modifications have potential for improving hydrophobicity of biopolymer films.

## 1. Introduction

Corn bran (CB) is a byproduct of the corn milling industry resulting from either dry milling (DCB) or wet milling (WCB) operations. Dried distiller’s grains and soluble solids (DDGS) are a coproduct of ethanol production [1]. CB includes beneficial constituents for the production of food additives containing protein, ferulic acid, and dietary fibers [2,3]. CB’s chemical composition is mainly made up of dietary fibers, with a higher proportion of insoluble fiber and a much smaller proportion of soluble fiber [4,5,6]. The main dietary fiber in CB is arabinoxylan (AX), with a smaller quantity of the dietary fiber cellulose. In addition to CB, DDGS are the most important coproduct from ethanol production in both amount and value, and DDGS are regularly utilized as an animal feed owing to their high sugar and protein contents [1,7,8].

CB and DDGS can have a substantial economic value and global impact on food supply, for both human nutrition and animal feed. The improvement of profits for corn processing facilities means using AX obtained from the DCB, WCB, or DDGS to create food packaging materials, which could replace non-biodegradable plastics. However, films made from CB AX and DDGS AX with laccase and sorbitol are highly hydrophilic materials as opposed to the hydrophobic materials that are typically used for food packaging [9,10]. Therefore, lipase-aided surface modifications with vinyl acetate must be conducted to increase hydrophobicity of the AX films [11]. Due to water barrier requirements, it is important to improve the hydrophobicity of the AX films when developing biodegradable materials for use in food packaging.

AX is a complex polymeric molecule consisting of a linear series of D-xylanopyranosyl (Xyl*p*) residues attached by β-1,4 glycosidic bonds that include arabinose (Ara*f*) substituents [12,13,14]. Corn AX’s structure contains 4-O-methyl-D-glucuronic acid at C (O)-2 and Ara*f* substituents at the C (O)-2 and/or C (O)-3 positions of the xylose residues [15,16]. Ferulic acid (FA) represents the majority of the phenolic acids found in CB and is present at the C (O)-5 position of the Ara*f*. In addition, although FA is found in small amounts, FA has important impacts on the solubility, molecular weight, and gel capacity of AX in the CB and after separation [12,17,18].

The use of AX as the foundation for packaging materials could become one source of biodegradable materials to produce hydrophobic films that may possibly be commercialized. A film made from AX is usually brittle with low extensibility and flexibility, initiating fragmentation when the film is handled. A plasticizer, sorbitol, is exploited to decrease brittleness and improve the flexibility of the AX films. Sorbitol is a practical plasticizer for food packaging as it is recognized as safe, a humectant, and non-carcinogenic [19,20]. The AX film might be modified using vinyl acetate with a lipase enzyme catalyst to improve the film’s hydrophobicity [11]. The lipase tends to reduce the amount of vinyl acetate needed for the esterification reaction [11,21]. Lipase has a high amount of reaction specificity for hydrolyzing ester bonds, leading to the esterification of AX [22,23,24]. This lipase activity can create esterified carbohydrate residues in the AX, resulting in the development of biomaterial films [11].

Food packaging ought to be suitable for the specific product being stored by matching the water barrier and mechanical properties of the material requirements. Mechanical attributes of AX films need be able to maintain the integrity of food packaging materials [25,26]. In order to maintain the moisture balance of specific foods, water interaction properties of AX films are also critical. Water interaction characteristics of AX films must be low, namely moisture content, water vapor transmission rate, and water solubility, for elongating the shelf life of the packaged foods [9,27,28]. In contrast, contact angle and biodegradability should be high for hydrophobic packaging materials which will rapidly degrade after use [9,19,25].

Currently, most food is packaged in plastics that are expensive, unhealthy, and environmentally unfriendly. In addition, many of the non-biodegradable materials (polyolefins, polyesters, and polyamides) used to package food for widespread consumption are also expensive and unnatural materials [29]. Thus, the enhancement of the crosslinking method is a creative strategy to enhance the functionality and water interaction characteristics of biopolymer films [30,31]. The creation of biopolymer food packaging systems will help market needs and consumer preferences for healthy and safe food products and decrease the loss of the food industries [32]. Green packaging can be exploited to diminish the ecological influences of food packaging by using plant extracts and biodegradable materials, such as those from corn processing byproducts. AX polymers extracted from corn processing byproducts have benefits over other green packaging materials. The AX polymers can be extracted from the waste material of corn processing which improves sustainability in these industries. Additionally, some green packaging materials such as bio-based polyethylene are not biodegradable. Other bioplastics such as polylactic acid are biodegradable but require the use of harsh solvents [29]. The AX polymers on the other hand are both biodegradable and water soluble. The DCB, WCB, or DDGS films can lead to a green packaging approach with less water interaction between packaged food components.

The aims of this study were to use AX extraction to produce biodegradable film materials, to establish how the AX films interact with water and soil (aerobic biodegradation), and to evaluate if these properties are associated with the AX characteristics. Biodegradable food packaging film has been generated through the mixture of AX extraction, sorbitol, and laccase [33]; in this research, the AX films were modified via immersion in a vinyl acetate with lipase.

## 2. Materials and Methods

### 2.1. Materials and Milling

Dry-milled corn bran (DCB) and wet-milled corn bran (WCB) were furnished by Archer Daniels Midland (ADM, Decatur, IL, USA). The dried distiller’s grains with solubles (DDGS) was purchased from Elk Mound Seed Company (Elk Mound, WI, USA). Both WCB and DDGS samples were ground using a cyclone sample mill (UDY corporation, Fort Collins, CO, USA) to reduce the particle size. Each DCB, WCB, or DDGS sample had three replications.

### 2.2. Arabinoxylan Extraction

The extraction of the AX from the DCB, WCB, and DDGS samples was the same as that described according to the study of Alahmed and Simsek [34] and was performed in triplicate. The AX extraction procedure is concisely explained here including three main steps: defatting, acid–alkali extraction, and ethanol precipitation. Each sample was defatted with hexane (1/10; *w*/*v*) to remove excess oil [33]. Next, 50 g of the defatted sample was placed in 500 mL of 0.25 M hydrochloric acid and stirred at 45 °C for 2 h. Then, 3.0 M sodium hydroxide (100 mL) was added to the slurry, while stirring before heating for another 2 h. The solution was neutralized (pH 7) using HCl and centrifuged for 10 min at 4945 rpm. The supernatant was collected, and AX was precipitated with ethanol [35]. After ethanol fractionation, the precipitate (AX) was collected via centrifugation before drying at 50 °C in an oven.

### 2.3. Arabinoxylan Polymer Characterization

The arabinoxylan content and Ara*f* and Xyl*p* ratio (A/X) of AX samples was determined with High-Performance Anion Exchange Chromatography with Pulsed Amperometry (HPAEC-PAD) in triplicate. An aliquot of 1 mL 1 M HCl was added to 5–6 mg of each sample [36]. The samples were then heated at 100 °C for 1 h before cooling and neutralizing the samples using 1 mL of 1 M NaOH. Then, the hydrolyzed samples were filtered via a nylon syringe filter (0.2 μm), and the sugars were determined using HPAEC-PAD with a CarboPac PA20 column following the method of the previous paper [36]. The AX content of the samples was calculated according to Formula (1):

(1) $$AX\left(\%\right)=0.88\times (\%Xylp+\%Araf)$$

High-Performance Size Exclusion Chromatography-Multi Angle Laser Scatting-Refractive Index (HPSEC-MALS-RI) was used to analyze weight average molecular weights (Mw) and the polydispersity index (PI) [37]. The analysis was performed in triplicate. The instrument included a Shimadzu HPLC attached to a Wyatt Opti lab RI detector and Wyatt MALS Dawn Heleos II. The separation was accomplished using two columns (PL Aquagel-OH 40 and 60) linked in series. The flow rate was 0.5 mL/min, and double-distilled water with 0.05% sodium azide was utilized as the mobile phase. The AX was liquefied in 50 mM sodium nitrate (NaNO_3_) (2 mg/mL) for 16 h before the solution was filtered via a 0.45 μm syringe filter. The dn/dc value for the AX was 0.146 to determine the weight average molecular weight (Mw) and polydispersity index (PI) using Astra 6.1.6 software, and the Zimm plot was used for the light scattering model with a fit degree of 1.

### 2.4. Preparation of Surface-Modified Arabinoxylan Films

The procedure for casting AX films is mentioned in detail according to the former study of Alahmed and Simsek [34]; however, a brief description is provided here as well. The AX film was generated by dissolving the polymer in deionized water with D-sorbitol [33], with minor modifications. Film solutions were produced by creating a 2.7% (*w*/*v*) solution of AX and laccase from *Aspergillus* sp. (130 µL/2.7% AX film solution) [18]. The AX solution was mixed at 25 °C for 24 h before being incubated at 90 °C for 15 min in a water bath. D-Sorbitol was then added to the AX solutions at 25% (*w*/*v*) [9,33] and heated for 15 min at 90 °C.

**Figure 1:**
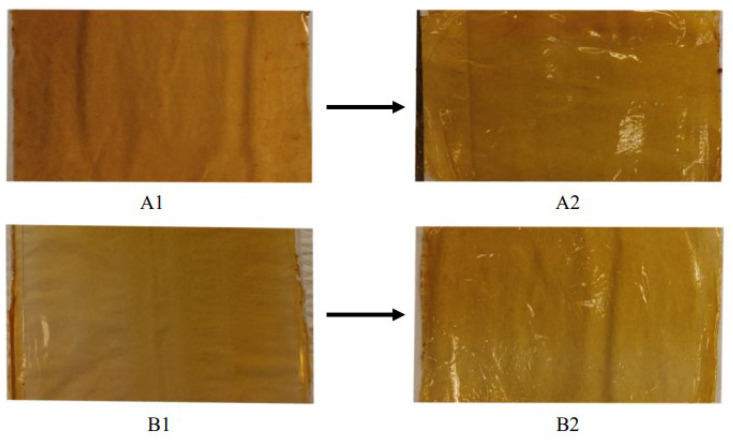
Representative example of unmodified dry milled corn bran arabinoxylan (**A1**), surface modified dry milled corn bran arabinoxylan (**A2**), unmodified wet milled corn bran arabinoxylan (**B1**), and surface modified wet milled corn bran arabinoxylan (**B2**) films prepared via pour casting. Note: Surface-modified with a lipase–vinyl acetate solution; Representative samples were chosen based on color and uniformity from all replicates after visual inspection under a controlled light source.

The liquid AX film solution was poured onto a square petri dish and dried overnight in a forced air oven at 60 °C. Once dried, all AX films were maintained at 25 °C and 50–60% relative humidity in a desiccator cabinet. Each unmodified film from each corn byproduct material included three replications (Figure 1A1,B1). Each modified AX film also had three replications (Figure 1A2,B2). The surface modification did not cause any visual differences in the film appearance (Figure 1). In order to increase hydrophobicity of the AX films, the surfaces of the DCB AX, WCB AX, and DDGS films were immersed into a lipase–vinyl acetate solution [11,34]. The modified AX films were suspended in the reagent mixture for 24 h at 40 °C in an incubator [11]. After 24 h, the film samples were washed three times with methanol and three times with hexane before being dried under the fume hood for 10 min. Finally, both modified and unmodified AX films were stored at 50–60% RH and 25 °C until water interaction and biodegradability analysis.

### 2.5. Hydrophobic and Biodegradable Arabinoxylan Films

#### 2.5.1. Moisture Content

The moisture content of each AX film was measured gravimetrically in a forced air oven [38]. The moisture determination was completed in triplicate. To start the moisture procedure, the initial weight of each AX film was recorded. Next, the film was heated at 130 °C for 1 h in an air oven (VWR^®^ Forced Air Ovens, Radnor, PA, USA). After 1 h, the film was cooled in the desiccator for 30 min, and then the final weight of each film was recorded. Thus, the moisture content of the AX film was determined with Equation (2):

(2) $$Film\;moisture\;content\;(\%)=\frac{Initial\;film\;mass-Final\;film\;mass}{Initial\;film\;mass}\times 100$$

#### 2.5.2. Water Solubility

The water solubility of the AX film was established with triplicate measurements according to previous studies [9,39]. Firstly, each film was cut into a rectangle (2 cm × 3 cm), and the initial weight of AX film was recorded. Next, the piece of film was placed into a Fisher brand plastic centrifuge tube, and 40 mL of distilled water was poured in the capped centrifuge tube. The tubes were shaken at 25 °C and 70 rpm for 1 h using a Lab Shaker Rocker. Then, each film–water solution was filtered through filter paper using a vacuum. The remaining solid material was dried at 25 °C overnight in the fume hood. A day later, the filter papers were folded and dried at 60 °C for 6 h in a forced air oven. The final weight of insoluble film was determined, and the percentage of water solubility was calculated using Equation (3):

(3) $$Water\;solubility\;(\%)=\frac{Initial\;film\;mass-Final\;film\;mass}{Initial\;film\;mass}\times 100$$

#### 2.5.3. Water Vapor Transmission Rate

The water vapor permeability experiment had two measurements: the water vapor transmission rate (WVTR) and water vapor permeance (WVP), which were determined by following the ASTM E96/E96M-22a^ε1^ [40]. The determination of these values was carried out in triplicate. The water method was utilized, and the testing condition was at 22 °C and 50–60% RH for each modified and unmodified AX film. During testing, the specimen weights were determined with an analytical balance scale, recording the weight at 0, 30, 60, 90, 120, 150, 180, 210, and 240 min, including 24 and 48 h [9]. The tools of the test contained the following: 10 mL distilled water, a 15 × 60 mm polystyrene petri dish, and two steel washers (with inner diameters of 3.2 cm and outer diameters of 5.7 cm) to hold the 3 cm × 4 cm AX film piece. The parafilm was used to secure the sample, petri dish, and steel washers to seal the entire apparatus. Equations (4) and (5) below were used to measure WVTR and WVP, respectively.

(4) $$WVTR\;(gh^{-1}m^{-2})=\frac{G}{tA}=\frac{\left(G/t\right)}{A}$$

where G = weight change in grams, t = time which G happened in hours, G/t = slope of G per h, and A = test area (inner diameter mouth area). In this study, A = 8.04 cm^2^.

(5) $$WVP\;(g/sm^{2}Pa)=\frac{WVTR}{△p}=\frac{WVTR}{S\left(R^{1}-R^{2}\right)}$$

where WVTR = water vapor transmission rate (g h^−^^1^ m^−2^), ∆p = vapor pressure difference in mm Hg (1.333 × 10^2^ Pa), S = saturation vapor pressure at test temperature (22 °C) in mm Hg (1.333 × 10^2^ Pa), R_1_ = relative humidity at the source measured with a hydrometer in the dish, and R_2_ = relative humidity at the vapor sink measured with a hydrometer outside of the dish. In this study, S = 19.84, R_1_ = 1, and R_2_ = 0.619702.

#### 2.5.4. Contact Angles

The contact angle was determined using distilled water by dispensing one drop of the water on each AX film following ASTM D7490-13 [41]. The testing condition was at 50–55% relative humidity and 25 °C for each modified and unmodified AX film. The measurement of contact angles was performed in triplicate with a Dynamic Contact Angle Analyzer including a charge coupled device camera [9]. One drop of distilled water was placed on the surface of each AX film using a syringe, and the camera rapidly took a picture of the drop’s angles. The contact angles were measured with the ImageJ software program (Version 1.54d) in the range from 0° to 180° based on the angles of the drop.

#### 2.5.5. Biodegradability Analysis

The biodegradability analysis for each modified and unmodified AX film was performed in two replicates using soil under alkaline conditions [9]. This testing was conducted by the following official method, ASTM D5988-18 [42]. Briefly, 100 g of soil was added into a 12 oz Clear Round Wide-Mouth Plastic Jar. Then, 300 mg of the AX film was put in the middle of the soil in the jar, and the remaining soil was used to cover the film. An uncapped clear round wide-mouth plastic vial (4 oz.) with 1 N NaOH (20 mL) was set on top of the soil, and the jar containing the soil and plastic vial was sealed tightly. A control specimen with no film and soil was used as a blank. The biodegradability is presented as the means of two independent replicates (*n* = 2). All jars were stored out of the light at 23–25 °C. Then, carbon and carbon dioxide balance (C-CO_2_) production was determined over a span of 168 days. The vial of NaOH was taken from the soil–film jars on days 6, 12, 19, 26, 33, 41, 63, 99, and 168 and replaced with a new vial of fresh NaOH solution. Then, 3 drops of diluted phenolphthalein were added to the NaOH, which was titrated using a 1 N HCI solution until a pH of 7.73–7.78 was reached. The carbon content of each modified and unmodified AX film needed to be quantified via combustion analysis for determining the amount of biodegradability. Equations (6) and (7) below were applied to determine the C-CO_2_, leading to the calculation of the percentage of biodegradability of each modified and unmodified AX film.

(6) $$Release\;of\;C\;per\;100\;g\;CO_{2}=(A-B)(Acid\;Molarity)(Eq.g\;C\;{CO}_{2})$$

where A = the initial amount of NaOH utilized in the attached film in mL, B = the amount of HCI used in titration sample in mL, AM = the molarity of HCI in M, and Eq.g. C-CO_2_ = the equivalent gram of hemicellulose. In this study, A = 20 mL, AM = 1, and Eq.g C-CO_2_ = 6.

(7) $$Film\;biodegradability(\%)=\frac{{CO}_{2}\;soil\;with\;film-{CO}_{2}\;soil\;with\;outfilm\;(blank)}{mg\;C\;in\;film}\times 100$$

### 2.6. Statistical Analysis

Statistical analysis was completed with SAS version 9.4 (SAS Institute, Cary, NC, USA) [43]. The first factor was the source of the AX polymer (DCB, WCB, and DDGS), and the second factor was the modification of the films. Two-way effects model ANOVA to evaluate the main effects of source, modification, and their interaction was performed for each dependent variable. Mean separation was determined using Fisher’s least significant difference (LSD). A Cell Means model was used to evaluate differences among the interaction means, and LSD was utilized for mean separation. All tests were evaluated using the significance level of 0.05.

## 3. Results and Discussion

### 3.1. Arabinoxylan Polymer and Molecular Weight

Arabinoxylan (AX) extracts were analyzed for their A/X ratio, Mw, and PI, and presented significant (*p* < 0.05) differences between each of the three samples, as exhibited in Table 1. The extract yields from the DCB and DDGS were 23.30% and 23.01%, respectively, whereas WCB accounted for a higher yield of 25.39% (Table 1). The high yield from WCB can be ascribed to dissimilarities in processing used to produce each of the corn byproducts. Dry milling of corn splits the principal components of the kernel—endosperm, bran, and germ [44]. On the other hand, the process of wet milling separates the corn’s constituents into starch, protein fiber, and oil [45]. Finally, production of corn ethanol has further stages such as fermentation, distillation, and centrifugation, which results in further breakdown of corn biopolymers [1].

The AX concentration and the arabinose to xylose ratios (A/X) varied depending on the corn byproduct sources, namely DCB, WCB, and DDGS. For the extracts derived from DCB, WCB, and DDGS, the AX content was 76.60%, 77.64%, and 66.68%, respectively, as presented in Table 1. Moreover, the A/X ratios were different, with DCB having the lowest (1.02) and DDGS having the highest (1.32). These variances in AX content and structure between the three extracts were statistically significant (*p* < 0.05), emphasizing the dissimilarity among the samples.

The Mw of the AX extracts differed significantly (*p* < 0.05), with DCB AX having the largest Mw at 4,063,667 (Table 1). These values may indicate that an increase in AX concentration correlates with a higher Mw, a concept presented by a previous study [22]. The current study showed a relationship between the AX content and Mw of the extract derived from WCB and DDGS. However, a low A/X ratio results in low AX solubility, assuming an increase in Mw of AX [46]. The Mw of AX derived from DCB was greater as opposed to the others due to its lower A/X ratio with a decrease in solubility.

The PI for all samples were significantly (*p* < 0.05) distinct between each of the three AX extracts. The DCB AX presented the highest PI of 3.05, while the WCB AX had a PI of 2.37, and DDGS AX had the lowest PI of 1.80. This difference in PI underscores the heterogeneity found in the AX extracted from the various sources, highlighting the complexity and range of their structural features.

### 3.2. AX Film Moisture Content

The moisture content of a film influences the mechanical properties and the water interactions of a solid film. When the moisture content of AX films increases, they become hydrophilic. However, the decrease in the moisture content of the films may indicate that the AX films have become more hydrophobic [9]. The moisture content of the DDGS AX film (17.03%) was significantly (*p* < 0.05) higher than that of both WCB AX and DCB films, at 15.13% and 14.77%, respectively (Table 2). The films made with the DCB AX were the most hydrophobic, while the films made from the DDGS AX were the least hydrophobic. The moisture contents between the WCB and the DCB films were not significantly (*p* > 0.05) different.

There was a significant (*p* < 0.05) change in moisture content between the unmodified films and the films with the lipase–vinyl acetate surface modification. The average moisture content of modified AX films was 14.79%, which was significantly (*p* < 0.05) lower than the average moisture of 16.50% for the unmodified AX films, as noted in Table 2. The AX films immersed in the lipase–vinyl acetate solution resulted in a lower moisture content and thus an increase in AX film hydrophobicity.

The modification of AX films may cause a barrier between water and the hydrophilic nature of the AX chains, resulting in a reduction in the availability of the hydroxyl group (OH) and, in turn, a decrease in water interactions in the AX chains through hydrogen bonds. This outcome can lead to a decrease in the film moisture content, as has been shown in previous research [28,47].

**Figure 2:**
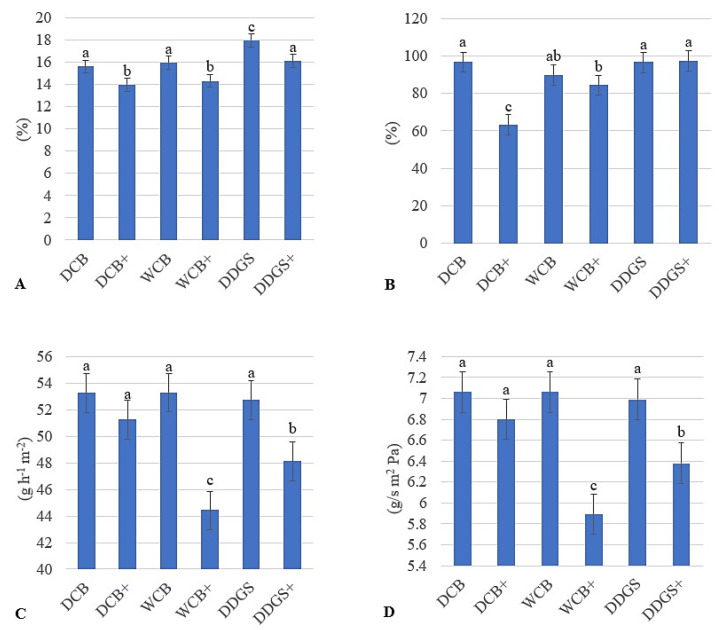
Water interactions of unmodified and modified AX films. (**A**) Moisture content. (**B**) Water solubility. (**C**) Water vapor transmission rate: WVTR. (**D**) Water vapor permeance: WVP. Bars in the same subfigure with the same letter are not significantly different (*p* > 0.05). The data are the means of three independent replicate experiments (*n* = 3). DCB: Dry corn bran; WCB: wet corn bran; DDGS: dried distiller’s grains with solubles; +: lipase–vinyl acetate surface modified film.

Interactions between a source and its modification affected the AX film moisture content. A significant (*p* < 0.05) decrease in moisture content was observed when the AX film surfaces were immersed in the lipase–vinyl acetate mixture. The moisture contents of all modified DCB AX, WCB AX, and DDGS films were significantly (*p* < 0.05) reduced compared to their unmodified AX film counterparts (Figure 2A).

Based on moisture content, the AX films suspended in a lipase–vinyl acetate solution were the most hydrophobic, while the unmodified AX films were the least hydrophobic materials. The tendency for increasing moisture content was due to the lack of lipase–vinyl acetate incorporation of the unmodified AX films. This is probably because the free OH group is available to interact with water molecules, leading to an AX film structural affinity for water uptake [28,47].

### 3.3. AX Film Water Solubility

Many foods already have high moisture contents, so packaging it in water-soluble materials is detrimental to both food safety and food quality. The DDGS films included the highest solubility in water, accounting for 96.94%, followed by WCB films with 87.08%, and DCB films with 79.92, as indicated in Table 2. Furthermore, the water solubility percentages of three AX films were significantly (*p* < 0.05) different from one specimen to another. As can be seen in Table 2, the low moisture content of the AX films produced low water solubility, leading to hydrophobic materials.

In this study, the AX component had an impact on the AX film water solubility. Previous work has shown that water solubility can be impacted by the AX structure. It has been demonstrated that A/X decreases as the AX structure increases in crystallinity, which leads to a lower water-solubility in AX films [48]. Again, in this study, the A/X ratio of the AX extract increased in the DDGS, thus the DDGS AX structure could theoretically impact the crystallinity and result in an increase in water solubility for the DDGS films, as seen above in Table 1 and below in Table 2. Conversely, the A/X of AX yields decreased in the DCB; therefore, the structure of AX tended to be more crystalline, leading to a decrease in solubility in water for the DCB films, as presented in both Table 1 and Table 2.

The water solubility was also evaluated for AX film surfaces which were immersed in a solution of lipase–vinyl acetate. A significant (*p* < 0.05) decrease in water-soluble film was observed for the modified DCB AX, WCB AX, and DDGS films as opposed to unmodified DCB AX, WCB AX, and DDGS films. The water solubility of modified DCB AX, WCB AX, and DDGS films was 81.61%, significantly (*p* < 0.05) lower than the 94.35% water solubility for unmodified DCB AX, WCB AX, and DDGS films (Table 2). Although the modified films were still highly soluble, the decrease in solubility shows that the surface modification has promise for the development of insoluble AX films.

A relationship between an increase in the value of the water solubility of the AX films and an increase in the existence of disubstituted xylose could be because of more spaces between the polymers and water [46]. After arabinose units are removed, disubstituted xylose is left. Then, water enters and dissolves the materials, leading to the high solubility of AX films [9,46]. Submerging modified AX films in a lipase–vinyl acetate solution resulted in a low water solubility, which in turn leads to more hydrophobic films and increased structural strength of food packaging materials.

The statistical interaction between the water solubility of the modified DCB films was significantly (*p* < 0.05) decreased compared to their unmodified AX film counterparts (Figure 2B). The water solubility for modified WCB films was reduced but was not significantly (*p* > 0.05) different from their unmodified AX films (Figure 2B). This decrease in the water solubility for both modified AX films means that they may be appropriate as hydrophobic materials for food packaging. A film’s low solubility and high hydrophobicity are essential characteristics that indicate more water tolerance for food packaging materials [28].

There was a correlation between film morphology and film water solubility that indicated cracks may be an entryway for water into the polymer matrix, leading to the film dissolving. Research bears out these results of the AX film morphology and water solubility [28]. The modified DCB AX and WCB films had lower surface fissures, resulting in lower water solubility for both modified DCB AX and WCB films compared to their unmodified AX films (DCB AX and WCB film morphology pictures not shown) [34]. The water solubility of the modified DDGS films remained unchanged as opposed to the unmodified DDGS AX film counterparts (Figure 2B). This is possibly attributable to the hydrophilic character of the DDGS films. Higher water solubility influences the commercial purpose of the packaging materials because aqueous food can destabilize the film [39].

### 3.4. Water Vapor Transmission Rate

WVTR is the continual flow of water vapor over time through the area of flat material under a specific temperature and humidity between two parallel surfaces. WVP is the time rate of the water vapor transmission via the surface area of the flat material generated by the difference in vapor pressure between two specific surfaces. A timed water method measured the vapor movement rate (every 30 min for 4 h, then at 24 h and 48 h) through the AX films. Because the 48 h measurement is the most effective for confirming the evaluation of WVTR and WVP, the following results section focuses on the measurement of water vapor permeability at 48 h. The thickness of the films tested in this work ranged from 88.9 to 104.1 μm [34]. It is important to note that the thickness of the films will play a role in the WVTR, and thicker films may have slower WVTR. This work produced thin flexible films which may be suitable as a wrapping material, thus the films were quite thin. Production of thicker films from this material for testing of WVTR may be valuable for future studies.

From the lowest to highest, the AX films’ WVTR from WCB, DDGS, and DCB sources were 48.85, 50.42, and 52.24 g h^−1^ m^−2^, respectively (Table 2). The difference between the WVTR of the WCB films and the DDGS films was not significant (*p* > 0.05); however, both were significantly (*p* < 0.05) lower than the WVTR of the DCB films, as outlined in Table 2. Similarly, the WVP of the WCB AX, DDGS AX, and DCB films were 6.47, 6.68, and 6.92 g/s m^2^ Pa, respectively (Table 2). The difference between the WVP of the WCB films and the DDGS films was insignificant (*p* > 0.05); however, both were significantly (*p <* 0.05) decreased from the WVP of the DCB films, as exhibited in Table 2.

As shown in the water vapor results, the WCB films were the least permeable to water vapor; thus, these hydrophobic films have a good potential for future biodegradable materials. In fact, films low in WVTR and WVP are hydrophobic substances, thus minimalizing their interactions with water [26]. In contrast, the DCB films were the most permeable to water vapor, resulting in hydrophilic and hygroscopic films. Films with higher water vapor permeability may be useful for applications where movement of moisture out of the package is important.

The increase in WVTR and WVP can be accredited to hydrophilic nature of AX and the heterogeneity of the AX films made from several sources [28,49]. Water vapor permeability could also be increased due to the random irregularities, such as bubbles and voids, during the film casting and drying process. These irregularities allow water to enter through films, resulting in decreased AX matrix molecular density which in turn facilitates more water vapor diffusion and more water interactions [28,49].

The addition of the lipase–vinyl acetate solution for the surfaces of the AX films was followed by decreases in both WVTR and WVP since the incorporation of the lipase–vinyl acetate mixture can hinder water molecules and decrease water vapor transport. Thus, the WVTR of the unmodified AX films was 53.08 g h^−1^ m^−2^, significantly (*p* < 0.05) decreased to 47.93 g h^−1^ m^−2^ for the modified AX films, as presented in Table 2. Correspondingly, the WVP of the unmodified AX films was 7.03 g/s m^2^ Pa, significantly (*p* < 0.05) decreased to 6.35 g/s m^2^ Pa for the modified AX films (Table 2). These decreases in WVTR and WVP may be linked to an increased hydrophobicity [26] as a result of the surface modification.

It is likely that the additional lipase–vinyl acetate mixture could enhance the moisture barrier characteristics of the modified AX films. Materials with low WVTR and WVP are more resistant to moisture transmission between packaged food and the adjoining atmosphere, reducing microbial spoilage and prolonging the food shelf life [27,28]. There was a significant (*p* < 0.05) correlation between thickness and water vapor permeability [34]. An increase in thickness in the modified AX films led to a decrease in both the WVTR and WVP of the modified AX films (thickness results not shown).

The overall result of the interactions in WVTR and WVP was observed for all three types of AX films. The WVTR and WVP of modified WCB and DDGS films were significantly (*p* < 0.05) reduced, as opposed to their unmodified film counterparts (Figure 2C,D). However, the WVTR and WVP of modified DCB films were also decreased, but (*p* > 0.05) insignificantly, compared to the unmodified DCB films (Figure 2C,D). It is most feasible that the increases in the thicknesses of both modified DCB AX and modified DDGS films [resulted in decreases in their WVTR and WVP in this research.

As film thickness increases, water vapor permeability decreases creating an equilibrium water pressure on the interior surface of the films [39]. Hydrophobicity was significantly (*p* < 0.05) increased with immersing AX films in a lipase–vinyl acetate solution made from WCB and DDGS, while the modified DCB films seemed to be hygroscopic materials. The reduction in WVTR and WVP in the AX films suspended in lipase–vinyl acetate could be the result of the AX polymer chains becoming less mobile, resulting in less water vapor diffusion, leading to low water vapor permeability. This assumption was in agreement with the water vapor permeability measurement reported [47].

### 3.5. Contact Angle

The contact angles inform us about the hydrophobicity of the solid surface being analyzed. Due to the polarity of water, films have a high degree of content angles, resulting in higher hydrophobic and lower hydrophilic materials appropriate for food packaging [28]. The WCB films were the most hydrophobic with average contact angles of 109.46°; the DDGS films were the next most hydrophobic with an average contact angle of 108.87°; the DCB films were the least hydrophobic with an average contact angle of 106.11° (Table 3). Therefore, the contact angle of all films was not significantly (*p* > 0.05) different from one sample to another.

Previous work has shown that there was an important correlation between the A/X ratio of the AX extract and the contact angle of the AX films with water [9]. As the A/X ratio of the AX extract decreased in the DCB, the disubstituted xylose or branching on the AX polymer increased and the DCB films became more hydrophilic (see above Table 1 and below Table 3). A decrease in the AX substitution can lead to an increase in the hydrophobicity of the AX films [9].

For unmodified and modified AX films, the contact angle changed minimally due to the surface modification with the vinyl acetate. The contact angle of unmodified AX films was 109.86°, not significantly (*p* > 0.05) higher than the contact angle of 106.43° for the modified AX films, as noted in Table 3.

**Figure 3:**
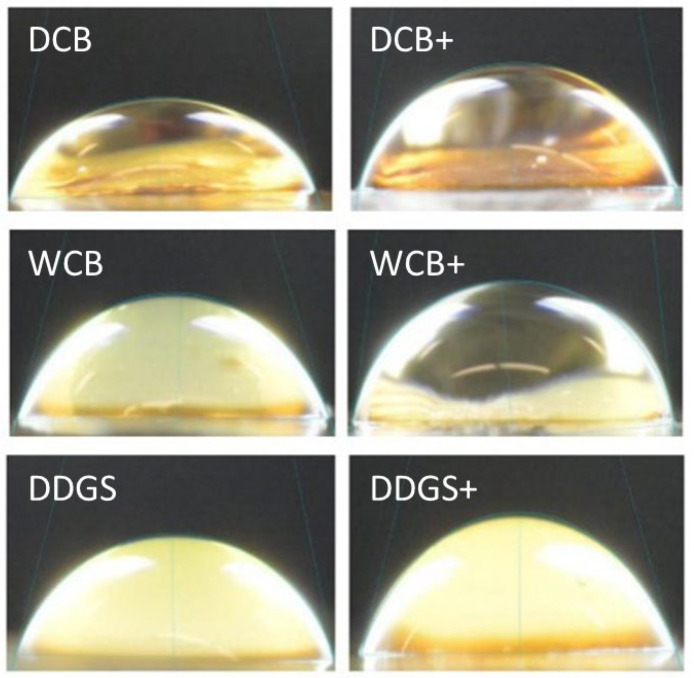
Contact angle pictures of unmodified and modified AX film samples. DCB: Dry corn bran; WCB: wet corn bran; DDGS: dried distiller’s grains with solubles; +: Lipase–vinyl acetate surface modified AX film.

Water was used to determine contact angle because the contact angle is very sensitive to surface contamination [41]. Thus, like other studies, the contact angle precision in this study was not recorded via laboratory testing [25]; however, for this study, the measurement of the contact angle on a solid surface was based on general disciplinary practice. Figure 3 provides the pictures of contact angles of the unmodified and modified AX films; it was challenging to precisely measure the contact angles and wettability of these AX films.

When the hydrophilicity of plant films improves, the capacity of the water absorption for the film seems to be increased [39]. Depending upon the contact angle results, the hydrophobicity of the AX-based films suspended in the lipase–vinyl acetate solution reduced, which tended to increase the water absorption capacity of the modified AX films.

The contact angle value of modified DCB films was 105.15° but was not significantly (*p* > 0.05) reduced as opposed to the contact angle of 107.08° for their unmodified film counterparts, as noted in Table 3. Similarly, the contact angle value of modified WCB films was 103.23°, but it insignificantly (*p* > 0.05) decreased compared to the contact angle of 115.70° for their unmodified AX films, as observed in Table 3. This suggests that the suspended lipase–vinyl acetate AX film surfaces were more hydrophilic with a higher affinity for water than their unmodified film surfaces, based on the contact angle results.

It is most likely that both modified DCB and WCB films had smooth surfaces, which may be an indicator for their hydrophilic films (modified DCB and modified WCB films morphology pictures not shown). The film roughness or smoothness plays an essential role in the precision of contact angle measurement and water interaction between particles [28].

In contrast, the contact angle value of modified DDGS films was 110.92° but increased with no (*p* > 0.05) significant difference compared to the contact angle of 106.81° for their unmodified film counterparts, as indicated in Table 3. The surface of DDGS films was affected by their suspension in the lipase–vinyl acetate mixture; thus, the modified DDGS films were hydrophobic with a lower affinity for water. Since a contact angle with >90° assumes a low wettability of a film’s surface [28], all AX films in this study were clearly hydrophobic materials with low wetting tendencies.

### 3.6. Biodegradability

Soil is a species-rich source of inoculum for the determination of biodegradability of packaging materials. Also, the biological activity of soil is considerable under alkaline conditions when oxygen availability is maintained. In this study, the biodegradability of AX films was assessed by determining the amount of CO_2_ released over a period of 6 months during which AX films were incubated in sandy soil. C-CO_2_ content of all AX films is presented in Table 3, showing no significant (*p* > 0.05) differences regarding the three factors: sources, modification, and interaction.

The WCB films were found to be the most biodegradable material with an average of 36.09%, followed by DCB films accounting for 31.82%; however, the DDGS films were the least biodegradable material at 28.91% (Table 3). Consequently, the biodegradability of WCB films was significantly (*p* < 0.05) different from the biodegradability of DDGS films.

There was a critical association between the hydrophobicity and the biodegradability of the AX-based films. As the WCB films had the highest hydrophobicity based on the contact angle results, the WCB films showed the greatest biodegradability amongst all samples, as illustrated in Table 3.

A rise in the heterogeneity of the AX polymers can provide a decrease in the biodegradability of the AX films due to the lack of the microbial breakdown of the films [9]. Similarly, the DDGS films included the lowest biodegradability as the AX polymers from DDGS had more ingredients (proteins, lipids, and free sugars) which are more heterogenous in the AX films made from DDGS.

No improvement was found in the biodegradability when the surfaces of AX films were submerged in the lipase–vinyl acetate solution. The percentage of biodegradability of the modified AX films was 32.24% but was not significantly (*p* > 0.05) different from the quantity of biodegradable material at 32.31% in the unmodified AX films, as outlined in Table 3. This outcome demonstrates that both unmodified and modified AX films were biodegradable and ecological amicable; this can facilitate low toxicity in the materials used for food packaging. The film biodegradation is associated with the film water solubility as higher solubility leads to greater biodegradation of films by the microbial organisms in the soil [50]. Although, based on the AX film sources in this research, the water solubility and biodegradability of the AX films results did not support this correlation.

**Figure 4:**
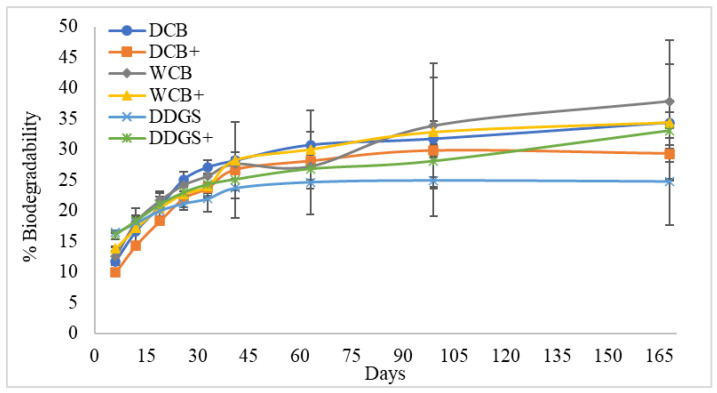
Biodegradability curves for unmodified and modified AX films prepared from extracts of corn bran from dry-milling (DCB), corn bran from wet-milling (WCB), and dried distiller’s grains with solubles (DDGS). +: Lipase–vinyl acetate surface modified AX films. Error bars represent standard deviation.

This study also found that the higher the biodegradability, the greater is the water solubility of the WCB and DDGS films when their surfaces were immersed in the lipase–vinyl acetate solution (see above Figure 2B and Table 3). It is most likely that the modified WCB AX and modified DDGS films increased in the rate of biodegradability because their water solubilities were not significantly (*p* > 0.05) changed when compared to the water solubility of their unmodified AX film counterparts. (see above Figure 2B and below Figure 4). It was discerned that the hydrophilic nature of AX polymers of WCB and DDGS improved the rates of biodegradation of the surface films when immersed in a lipase–vinyl acetate mixture.

In contrast, the biodegradation of modified DCB films did not increase within 168 days compared to the unmodified DCB films, as found in Figure 4. Interestingly, the water solubility of modified DCB films significantly decreased (*p* < 0.05) from the unmodified AX films, leading to lower biodegradability (see above Figure 2B and see below Figure 4).

The amount of film biodegradation is related to the film solubility in water, which means that lower solubility reduces the biodegradability of films [50].The AX films in this research were generally biodegradable even after modification of the their surfaces with the lipase–vinyl acetate solution.

## 4. Conclusions

This study has effectively demonstrated that modified arabinoxylan (AX) films, specifically those developed from DCB, WCB, and DDGS, exhibit significantly reduced moisture content, water solubility, and vapor permeability after treatment with a lipase–vinyl acetate solution. These modifications render the films more hydrophobic, improving their utility as eco-friendly food packaging materials that contribute to extended shelf-life and reduced environmental impact, aligning with global sustainability goals [18,51].

The biodegradability of these modified films, particularly from WCB and DDGS, demonstrated an enhanced degradation process compared to their unmodified counterparts [52]. This indicates that the hydrophilic nature of AX polymers from WCB and DDGS not only supports the biodegradation process but also improves the environmental friendliness of these films.

Future studies should focus on optimizing the film modification process for better scalability and economic viability. Additionally, further research could explore the effectiveness of these biodegradable films under different environmental conditions to fully assess their performance and durability. Performing comprehensive life cycle assessments of these films could provide invaluable insights into their overall environmental impacts compared to traditional packaging materials.

Furthermore, given the low oxygen gas permeability exhibited by these AX films as suggested by the American Society for Testing and Materials (ASTM) Method D3985-17 [52], it would be prudent to investigate their potential as oxygen barriers in various packaging applications. This could significantly reduce oxidation and spoilage in packaged foods, further enhancing food safety and quality.

In summary, the development of hydrophobic and biodegradable AX films offers a promising solution to the challenges posed by non-biodegradable packaging materials. The potential of these films extends beyond food packaging, as they could be adapted for use in pharmaceutical packaging and other applications where barrier properties are crucial. Collaboration among researchers, industry stakeholders, and policymakers is essential to explore and expand the adoption of this technology, ensuring a sustainable future for packaging practices.

## Figures and Tables

**Table 1:** Characteristics of the arabinoxylan content derived from three sources.

	Yield (%, DWB)	AX Content (%, DWB)	A/X	Mw	PI
DCB	23.30 b	76.60 b	1.02 c	4,063,667 a	3.05 a
WCB	25.39 a	77.64 a	1.20 b	1,876,333 b	2.37 b
DDGS	23.01 b	66.68 c	1.32 a	879,133 c	1.80 c

Note: Means with the same letter in the same column for the same sample type are not significantly different (*p* > 0.05). The data are the means of three independent replicate experiments (*n* = 3). Dry corn bran: DCB; wet corn bran: WCB; dried distiller’s grains with solubles: DDGS. A/X: arabinose to xylose ratio; Mw: weight average molecular weight; PI: polydispersity index.

**Table 2:** Water interactions of unmodified and surface-modified AX films.

Factors	AX Films	Moisture Content (%)	Water Solubility (%)	WVTR g h^−1^ m^−2^	WVP g/s m^2^ Pa
Sources	DCB	14.77 b	79.92 c	52.24 a	6.92 a
	WCB	15.13 b	87.08 b	48.85 b	6.47 b
	DDGS	17.03 a	96.94 a	50.42 b	6.68 b
Modification	Unmodified	16.50 a	94.35 a	53.08 a	7.03 a
	Modified	14.79 b	81.61 b	47.93 b	6.35 b

Note: Means with the same letter in the same column for the same factor are not significantly different (*p* > 0.05); The data are the means of three independent replicate experiments (*n* = 3); Dry corn bran: DCB; wet corn bran: WCB; dried distiller’s grains with solubles: DDGS.

**Table 3:** Hydrophobicity and biodegradability of unmodified and modified AX film samples.

Factors	AX Films	Contact Angle (°)	C-CO_2_ (mg)	Biodegradability (%)
Sources	DCB	106.11 a	9.84 a	31.82 ab
	WCB	109.46 a	12.43 a	36.09 a
	DDGS	108.87 a	11.73 a	28.91 b
Modification	Unmodified	109.86 a	11.45 a	32.31 a
	Modified	106.43 a	11.22 a	32.24 a
Interactions	DCB	107.08 a	12.27 a	34.35 ab
	DCB+	105.15 a	7.41 a	29.30 bc
	WCB	115.70 a	14.25 a	37.82 a
	WCB+	103.23 a	10.62 a	34.36 ab
	DDGS	106.81 a	7.83 a	24.75 c
	DDGS+	110.92 a	15.63 a	33.06 ab

Note: Means with the same letter in the same column for the same factor are not significantly different (*p* > 0.05). The data of contact angle are the means of three independent replicates (*n* = 3). The data of biodegradability are the means of two independent replicates (I = 2). DCB: Dry corn bran; WCB: wet corn bran; DDGS: dried distiller’s grains with solubles; +: Lipase–vinyl acetate surface modified AX film.

## Data Availability

The original contributions presented in the study are included in the article, further inquiries can be directed to the corresponding author.
